# Towards a Systems Biology Approach to Understanding the Lichen Symbiosis: Opportunities and Challenges of Implementing Network Modelling

**DOI:** 10.3389/fmicb.2021.667864

**Published:** 2021-05-03

**Authors:** Hadi Nazem-Bokaee, Erik F. Y. Hom, Andrew C. Warden, Sarah Mathews, Cécile Gueidan

**Affiliations:** ^1^CSIRO Australian National Herbarium, Centre for Australian National Biodiversity Research, National Research Collections Australia, NCMI, Canberra, ACT, Australia; ^2^CSIRO Land and Water, Canberra, ACT, Australia; ^3^CSIRO Synthetic Biology Future Science Platform, Canberra, ACT, Australia; ^4^Department of Biology and Center for Biodiversity and Conservation Research, The University of Mississippi, University City, MS, United States; ^5^Department of Biological Sciences, Louisiana State University, Baton Rouge, LA, United States

**Keywords:** systems biology, network modelling, signalling, metabolic model, lichen symbiosis

## Abstract

Lichen associations, a classic model for successful and sustainable interactions between micro-organisms, have been studied for many years. However, there are significant gaps in our understanding about how the lichen symbiosis operates at the molecular level. This review addresses opportunities for expanding current knowledge on signalling and metabolic interplays in the lichen symbiosis using the tools and approaches of systems biology, particularly network modelling. The largely unexplored nature of symbiont recognition and metabolic interdependency in lichens could benefit from applying a holistic approach to understand underlying molecular mechanisms and processes. Together with ‘omics’ approaches, the application of signalling and metabolic network modelling could provide predictive means to gain insights into lichen signalling and metabolic pathways. First, we review the major signalling and recognition modalities in the lichen symbioses studied to date, and then describe how modelling signalling networks could enhance our understanding of symbiont recognition, particularly leveraging omics techniques. Next, we highlight the current state of knowledge on lichen metabolism. We also discuss metabolic network modelling as a tool to simulate flux distribution in lichen metabolic pathways and to analyse the co-dependence between symbionts. This is especially important given the growing number of lichen genomes now available and improved computational tools for reconstructing such models. We highlight the benefits and possible bottlenecks for implementing different types of network models as applied to the study of lichens.

## Introduction

Lichens are often seen as a typical example of successful and sustainable symbiotic interactions between micro-organisms (Ahmadjian, 1993; Honegger, 1998). With the long evolutionary history of these fungal-algal associations (Gueidan et al., 2011; Prieto and Wedin, 2013; Lutzoni et al., 2018; Nelsen et al., 2019) and their multiple origins within the evolution of fungi (Gueidan et al., 2008; Schoch et al., 2009; Nelsen et al., 2020), lichens have colonised and diversified greatly in most terrestrial and some aquatic environments, including the most inhospitable niches (Kappen, 2000; Sadowsky and Ott, 2016). They are a discrete but inherent part of most of our landscapes, including both natural and man-made. This success stems from their ability to act as self-sustainable ecosystems, for which an evolutionary modularity (i.e., selection of the most fitted partners for a particular environment) has allowed adaptation to a broad range of habitats. Because of their slow growth, they particularly excel in colonising harsh habitats in which competition with faster growing micro-organisms is low. As such, they have adapted to surviving on nutrient-poor substrates and under drastically fluctuating environmental conditions, and play key roles in their ecosystems. In the future, lichen adaptations and their natural ecological flexibility may prove to be key to the successful responses of lichens to climate change.

The lichen symbiosis is no longer perceived to be the simple union of a fungal partner (i.e., mycobiont) and a microalgal partner (i.e., photobiont), either an alga (i.e., chlorolichen) or a cyanobacterium (i.e., cyanolichen). Instead, previous studies have shown that lichens harbour a diverse microbiome (e.g., Petrini et al., 1990; Hofstetter et al., 2007; Grube et al., 2009; Hodkinson and Lutzoni, 2009), and more recent studies corroborate lichens as multi-symbioses, i.e., complex multi-species associations including bacteria and other fungi or algae (Spribille et al., 2016; Onut-Brannstrom et al., 2018; Tuovinen et al., 2019; Smith et al., 2020; Leiva et al., 2021). In such symbioses, each partner contributes to the association: the primary mycobiont provides shelter and minerals to the photobiont, while the photobiont provides organic carbon fixed from atmospheric CO_2_ *via* photosynthesis (Nash, 2008a) as well as nitrogen if it is a cyanobacteria. Additional bacteria, algae, and/or fungi have also been shown to serve certain functions in the lichen symbiosis (Cernava et al., 2017; Smith et al., 2020; Tagirdzhanova et al., 2021), although much more remains to be explored. Additionally, the levels of dependence and specificity of some of these microbes to the symbiosis are still debated (Grube et al., 2015; Kono et al., 2017; Jenkins and Richards, 2019; Lendemer et al., 2019; Smith et al., 2020). Lichens demonstrate unique physiological properties and ecosystem functions (Porada et al., 2014). All lichens contribute to atmospheric carbon fixation, with global net carbon uptake by both lichens and bryophytes predicted to be 0.34–3.3 Gt carbon per year (Palmqvist, 1995; Green et al., 2008; Palmqvist et al., 2008; Porada et al., 2013). Cyanolichens are capable of both carbon and nitrogen fixation (Dahlman et al., 2004; Nash, 2008b; Porada et al., 2017). Lichens grow on various substrates (including rocks, trees, and soil), can survive extreme temperatures, tolerate desiccation (poikilohydric) and high levels of UV radiation, and form morphologically diverse structures (Beckett et al., 2008; Kranner et al., 2008). Many lichens produce unique specialised/secondary metabolites, including depsides, xanthones and dibenzofurans, some of which have been shown to have medicinal properties (Fahselt, 1994; Elix and Stocker-Worgotter, 2008; Calcott et al., 2018).

**FIGURE 1:**
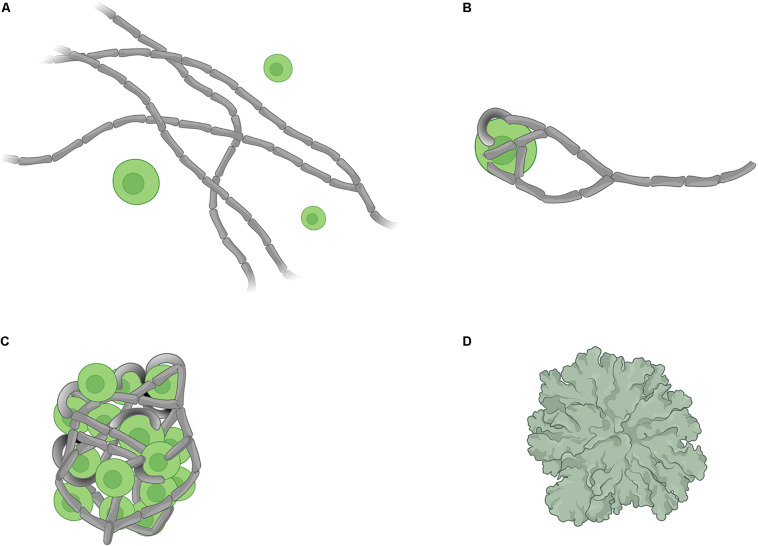
Schematic representation of the four stages of lichen formation. **(A)** pre-contact where the symbionts are located at the proximity of each other but not in physical contact, **(B)** post-contact which marks the initiation of physical contacts between symbionts, **(C)** growth of an undifferentiated mass consisting of fungal hyphae and algal cells, and **(D)** formation of a differentiated thallus. (Image created with BioRender.com).

The establishment of the lichen symbiosis, or “lichenisation,” has been described as a four-stage process (Ahmadjian et al., 1978): (A) a pre-contact phase (chemical interactions between symbionts but no physical contact), (B) a post-contact phase (with chemical and physical interactions), (C) a phase of growth characterised by an un-differentiated mass, and (D) a phase of differentiation that leads to a stratified thallus (Figure 1). Because mycobionts grow relatively slowly, the application of classical experimental microbiology techniques and co-culture/resynthesis experiments to the understanding of the development and functioning of the lichen symbiosis has lagged. Despite some recent studies focusing on early stages of lichenisation (Joneson et al., 2011; Armaleo et al., 2019; Kono et al., 2020), the molecular basis of fungal-algal interactions during lichenisation remains mostly uncharacterised, and processes involved in signalling and metabolic interplays between the symbionts are poorly understood. Contemporary systems biology approaches may facilitate tackling long-standing questions about the lichen symbiosis.

Systems biology is the study of living systems through the joint application of advanced high-data-volume generating technologies (e.g., ‘omics’) and computational tools (e.g., multi-scale or constraint-based modelling) to gain a more holistic understanding of the inter-dependencies of system components and underlying system complexity. Hypotheses are generally tested using iterative cycles of ‘wet’ (lab-based) and ‘dry’ (simulation-based) experiments, by which systems-level data are generated, analysed, and then used to inspire new insights and hypotheses about the biological system at hand (Kitano, 2002a, b). For instance, applying systems- and genome-level approaches to the legume-rhizobium symbiosis has greatly enhanced the knowledge on the underlying mechanisms of symbiotic interactions at molecular level, moving us one step closer to improving agricultural crop yields through the development of more efficient symbiotic N_2_ fixation processes (diCenzo et al., 2019). A similar systems biology approach has not yet been applied to the study of the lichen symbiosis.

In this review, we summarise the current knowledgebase of signalling and recognition mechanisms in the lichen symbiosis. We then discuss the modelling of signalling networks as a tool to extend our understanding of such mechanisms in lichens. We review the literature on lichen metabolism and propose that modelling fluxes in metabolic networks could be a powerful tool for providing insights into lichen metabolism in particular, and the metabolic interplays between symbiotic partners in general. We provide a broad overview of metabolic network models and their applications in addition to a review of some of the symbiotic systems that have been studied through the lens of metabolic models. Finally, the opportunities and challenges of modelling both signalling networks and metabolic fluxes are discussed.

## Signalling and Recognition Pathways in the Lichen Symbiosis

Distinct small molecules are produced by lichen symbionts during symbiosis that are absent when mycobiont and photobiont are grown separately (Green and Smith, 1974; Elshobary et al., 2015). Whether symbiont signalling and recognition processes in lichens are driven initially by those small molecules, or whether recognition processes are initiated by other regulatory mechanisms is not known. The available data for molecules with potential roles in signalling and/or recognition mechanisms during lichen symbiosis are summarised in Table 1. So far, there is no direct evidence confirming the production of compounds with a potential role in signalling and/or recognition during lichenisation by inhabiting fungi or bacteria. Several studies have shown that signalling between lichen symbionts can be initiated as early as the pre-contact stage of lichenisation (Joneson et al., 2011; Meessen and Ott, 2013; Piercey-Normore and Athukorala, 2017; Armaleo et al., 2019). At present and for a few reasons, it is difficult to propose universal signalling models that initiate lichen symbiosis. Firstly, there is no single signalling molecule with a known or proposed role that has been studied across different lichens. Secondly, signalling pathways of those molecules with putative recognition roles have not been elucidated. Thirdly, lichens have likely evolved independently in several fungal lineages (Gueidan et al., 2008; Schoch et al., 2009), suggesting that the nature of these signalling pathways might differ depending on the species of interest. Nonetheless, owing to advances in genetic and analytical tools, several studies have begun to uncover mechanistic details underlying partner signalling and recognition at various stages of lichenisation (Meessen et al., 2013; Meessen and Ott, 2013; Athukorala et al., 2014; Athukorala and Piercey-Normore, 2015).

**TABLE 1:** Molecules produced by different lichen symbionts with proposed roles in symbiotic signalling and recognition.

Molecule	Chemical class	Proposed role	Mycobiont	Photobiont°	References
**Produced by the mycobiont**					
Algal binding protein (ABP)	Glycoprotein	Plays a role in recognition of photobiont ligand	*Xanthoria parietina*^1^	*Trebouxia sp.?*	Molina et al., 1993; Molina and Vicente, 2000
Cyanobacterium-binding protein (CBP)	Possibly a glycoprotein	Plays a role in the first step of the recognition of compatible symbionts in a cyanolichen	*Peltigera canina*^2^	*Nostoc sp.*	Diaz et al., 2009
			*Scytinium palmatum*^4^	*Nostoc sp.*	Vivas et al., 2010
Galectin LEC-1 and LEC-2	Glycan-binding proteins	Plays a role in recognition of photobiont ligand	*Peltigera membranacea*^2^	*Nostoc sp.*	Manoharan et al., 2012; Miao et al., 2012
*Nephroma laevigatum* agglutinin (NLA)	Possibly a glycoprotein	Functions as a determinant of specificity at the initial stage of symbiont interaction	*Nephroma laevigatum*^3^	*Nostoc sp.*	Kardish et al., 1991
*Peltigera membranacea* agglutinin (PMA)	Glycoprotein	Functions in the recognition process between symbionts	*Peltigera membranacea*^2^	*Nostoc sp.*	Lehr et al., 1995
Phytohemagglutinins	Glycoprotein	May be involved in the initial stages of the symbiosis establishment	*Peltigera canina*^2^	*Nostoc sp.*	Lockhart et al., 1978
			*Peltigera polydactyla*^2^	*Nostoc sp.*	Lockhart et al., 1978
Phytolectin	Glycoprotein	May be involved in the recognition or initial interactions between compatible lichen symbionts	*Peltigera horizontalis*^2^	*Nostoc sp.*	Petit, 1982
			*Peltigera canina* var. *canina*^2^	*Nostoc sp.*	Petit et al., 1983
Secreted arginase of *Evernia* (SAE)	Hydrolytic enzyme	Plays a role in recognition of photobiont ligand (e.g., urease)	*Evernia prunastri*^5^	*Trebouxia excentrica*	Legaz et al., 2004
Secreted arginase of *Xanthoria* (SAX)			*Xanthoria parietina*^1^	*Trebouxia sp.?*	Molina et al., 1993; Molina and Vicente, 2000
			*Xanthoria parietina*^1^	*Pseudotrebouxia aggregata*	Legaz et al., 2004
*Xanthoria*-protein	Glycoprotein	May have role in initiation of lichen resynthesis and discriminate between photobionts	*Xanthoria parietina*^1^	*Trebouxia sp.*	Bubrick and Galun, 1980; Bubrick et al., 1981
			*Variospora aurantia*^1^	*Pseudotrebouxia sp.*	Bubrick and Galun, 1980
			*Flavoplaca citrina*^1^	*Pseudotrebouxia sp.*	Bubrick and Galun, 1980
**Produced by the photobiont**					
Chitinase	Hydrolytic enzyme	Regulates controlled parasitism between the symbionts	*Cladonia rangiferina*^6^	*Asterochloris sp.*	Athukorala and Piercey-Normore, 2015
Cyclo-L-leucyl-L-tyrosyl (CLT)	Cyclic dipeptide*	Not known	*Romjularia lurida*^8^	*Asterochloris sp.*	Meessen et al., 2013
Cyclo-L-tryptophyl-L-tryptophyl (CTT)	Cyclic dipeptide*	Promotes the germination rate of mycobiont *in vitro* after 30 days	*Gyalolechia bracteata* ^1^	*Trebouxia sp.*, Cl.1, sbgr.1	Meessen et al., 2013
		Not known	*Gyalolechia fulgens* ^1^	*Trebouxia sp.*, Cl.1, sbgr.1	Meessen et al., 2013
		Not known	*Thalloidima sedifolium* ^7^	*Trebouxia sp.*, Cl.1, sbgr.1	Meessen et al., 2013
		Not known	*Xanthoria elegans*^1^	*Trebouxia sp.*	Meessen et al., 2013
Indole-3-carbaldehyde (ICA)	Phytohormone precursor	Decreases the germination rate of mycobiont *in vitro*	*Gyalolechia bracteata* ^1^	*Trebouxia sp.*, Cl.1, sbgr.1	Meessen et al., 2013
		Not known	*Gyalolechia fulgens* ^1^	*Trebouxia sp.*, Cl.1, sbgr.1	Meessen et al., 2013
		Not known	*Thalloidima sedifolium* ^7^	*Trebouxia sp.*, Cl.1, sbgr.1	Meessen et al., 2013
		Not known	*Xanthoria elegans*^1^	*Trebouxia sp.*	Meessen et al., 2013
Rhamnose	Deoxy sugar	Decreases the germination rate of mycobiont *in vitro*	*Gyalolechia bracteata*^1^	*Trebouxia sp.*, Cl.1, sbgr.1	Meessen et al., 2013
Ribitol	Sugar alcohol	Acts as pre-/post-contact signal molecule	*Cladonia grayi*^6^	*Asterochloris sp.*	Joneson et al., 2011
		Overcomes the growth arrest of the mycobiont and promotes mycelium growth^#^	*Gyalolechia bracteata* ^1^	*Trebouxia sp.*, Cl.1, sbgr.1	Meessen et al., 2013
Urease	Hydrolytic enzyme	Serves as a ligand for different lichen lectins	*Xanthoria parietina*^1^	*Pseudotrebouxia aggregata*	Millanes et al., 2004
			*Evernia prunastri*^1^	*Trebouxia excentrica*	Millanes et al., 2004
**Produced by the symbiosis as a whole (The experiment settings did not allow to attribute the compound to either the mycobiont or the photobiont)**					
1-aminocyclopropane-l-carboxylic acid (ACC)	Phytohormone precursor	Affects differentiation and regulates interactions in the lichen thallus	*Cladonia rangiferina*^6^	Unidentified	Ott et al., 2000
		Not known	*Usnea longissima*^5^	Unidentified	Ott et al., 2000
		Not known	*Parmelia saxatilis*^5^	Unidentified	Ott et al., 2000
		Not known	*Usnea sphacelata*^5^	Unidentified	Ott et al., 2000
		Not known	*Peltigera polydactyla*^2^	Unidentified	Ott et al., 2000
		Not known	*Peltigera canina*^2^	Unidentified	Ott et al., 2000
		Not known	*Nephroma resupinatum*^3^	Unidentified	Ott et al., 2000
		Not known	*Scytinium palmatum*^4^	*Nostoc sp.*	Vivas et al., 2010
Abscisic acid (ABA)	Phytohormone	Affects differentiation and regulates interactions in the lichen thallus	*Cladonia rangiferina*^6^	Unidentified	Ott et al., 2000
		Not known	*Cladonia arbuscula*^6^	Unidentified	Ott et al., 2000
		Not known	*Cetraria islandica*^5^	Unidentified	Ott et al., 2000
Indole-3-acetic acid (IAA)	Phytohormone	Affects differentiation and regulates interactions in the lichen thallus	*Cladonia rangiferina*^6^	Unidentified	Ott et al., 2000
		Not known	*Peltigera hymenina*^2^	Unidentified	Ott et al., 2000
		Not known	*Cetraria islandica*^5^	Unidentified	Ott et al., 2000
		Not known	*Cladonia arbuscula*^6^	Unidentified	Ott et al., 2000
		Not known	*Ramalina duriaei*^7^	*Trebouxia sp.*	Epstein et al., 1986

°Trebouxia, Pseudotrebouxia, and Asterochloris are eukaryotic algae (Chlorophyta, Trebouxiophyceae) and Nostoc is a prokaryotic alga (cyanobacteria). Fungal lineages are as follows: ^1^ Ascomycota, Lecanoromycetes, Teloschistales, Teloschistaceae; ^2^ Ascomycota, Lecanoromycetes, Peltigerales, Peltigeraceae; ^3^ Ascomycota, Lecanoromycetes, Peltigerales, Nephromataceae; ^4^ Ascomycota, Lecanoromycetes, Peltigerales, Collemataceae; ^5^ Ascomycota, Lecanoromycetes, Lecanorales, Parmeliaceae; ^6^ Ascomycota, Lecanoromycetes, Lecanorales, Cladoniaceae; ^7^ Ascomycota, Lecanoromycetes, Lecanorales, Ramalinaceae; ^8^ Ascomycota, Lecanoromycetes, Lecideales, Lecideaceae. ^#^Ribitol in these pre-contact experiments (mycobiont and photobiont separated by a membrane) was added in concentrations of 0.05, 0.8, and 2.0%w/v to the culture media (water agar and malt yeast agar). Ribitol was not identified as photobiont exudate in these experiments, as seen in other studies (Richardson et al., 1968). *Belong to the class of diketopiperazines (DKPs) with potential applications as antitumor, antiviral, antifungal, and antibacterial properties.

### Lectin-Ligand Signalling in Lichens

**FIGURE 2:**
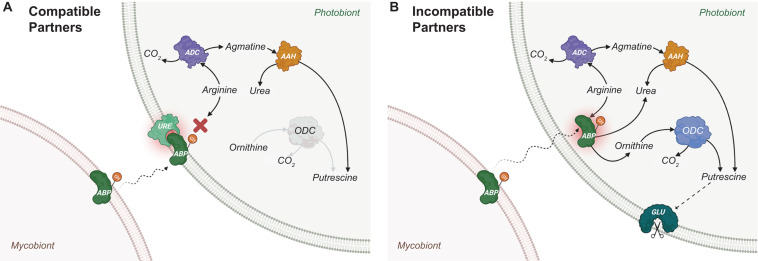
Proposed lectin-ligand recognition and signalling for *Xanthoria parietina.* **(A)** For compatible partners. The process starts by the production and positioning of algal binding protein (ABP) on the cell wall of the mycobiont. ABP is a lectin (“receptor”) which is glycosylated and secreted into the intercellular space (Molina et al., 1993; Molina and Vicente, 1995) to bind specific cognate ligands of a photobiont, with which a potential lichenisation could be established. It is speculated that ABP is part of a group of “recognition lectins” that is responsible for engaging the mycobiont with a specific photobiont, and another group of “recruitment lectins” that play a role in recruiting the photobiont cells to mycobiont hyphae (Sacristán et al., 2007). In the lichen *X. parietina*, a recruitment lectin was identified as a secreted arginase of the *Xanthoria* mycobiont (SAX). Although both ABP (recognition) and SAX (recruitment) mycobiont lectins were shown to bind to the ligand URE, a glycosylated urease located on the compatible photobiont cell wall (Millanes et al., 2004), it is not clear whether both must attach to urease for lichenisation to proceed. Both ABP and SAX lectins possess identical peptide sequences and Mn^2+^-dependent arginase activity, hydrolysing arginine to produce urea and ornithine (Legaz et al., 2004). However, the glycosidic moiety of SAX contains galactose and glucose, whereas that of ABP contains glucose and N-acetyl-glucosamine (Molina et al., 1993). Upon binding, enzymatic activities of ABP (recognition lectin and receptor) and URE (ligand) are inhibited. As a result, putrescine concentrations in a compatible photobiont are kept at relatively low endogenous levels because it is only produced through arginine decarboxylase (ADC) and agmatine amidinohydrolase (AAH). **(B)** For incompatible partners. The binding selectivity of ABP (receptor on mycobiont) for URE (ligand on photobiont) is the key in compatible partner recognition. For example, the ABP lectin receptor of *E. prunastri* can only bind to a single galactosylated ligand (Diaz et al., 2016c). Thus, an incompatible photobiont lacking galactosylated URE would not be recognised, resulting in an internalisation of ABP by the photobiont. Without URE ligand-induced inhibition, ABP goes on to hydrolyse cytoplasmic arginine stores in the photobiont (ABP has a higher affinity for arginine than that of ADC), producing urea and ornithine and a reaction cascade (involving both ornithine decarboxylase (ODC) and AAH) that results in increased cytoplasmic putrescine levels. High intracellular putrescine concentrations lead to the activation of glucanase (GLU), which results in cell wall disruptions and, ultimately, death of the incompatible photobiont cells (Molina et al., 1998). (Image created with BioRender.com).

Lectins are glycoproteins that occur ubiquitously across all domains of life (Kennedy et al., 1995). Lectins have also been isolated and characterised from both chlorolichens and cyanolichens (Table 1). Possessing versatile carbohydrate-binding site(s), lectins can act as receptors and/or bind/agglutinate cells that may facilitate further interfacial communication between cells. The glycosidic moieties of lectins synthesised by the mycobiont may contain various combinations of carbohydrate groups that bind to specific ligands from the photobiont. In this atypical receptor-ligand system, lectins from mycobionts act as receptors for photobiont-attached ligands. A proposed mechanism of photobiont recognition and recruitment by the mycobiont is illustrated in Figure 2, based on the extensive studies of the lichens *Xanthoria parietina* and *Evernia prunastri* (Bubrick and Galun, 1980; Bubrick et al., 1981; Perezurria and Vicente, 1989; Vicente and Perezurria, 1989; Rodriguez and Vicente, 1991; Molina et al., 1993, 1998; Molina and Vicente, 1995, 2000; Legaz et al., 2004; Millanes et al., 2004). Lectins characterised from other lichens also have been proposed to have roles in the establishment and/or maintenance of compatible symbiotic relationships (Table 1).

In several lichen associations (including *X. parietina* and *E. prunastri* shown in Figure 2), the ligand for lectin receptors has been identified as urease, which is bound to the cell wall of the photobiont (Molina et al., 1993; Millanes et al., 2004; Diaz et al., 2009). In the lichen *Cladonia rangiferina*, a urease-like recognition-related protein (RR1) was characterised and speculated to act as a ligand on the cell wall of the compatible photobiont of this lichen association (Athukorala et al., 2014; Athukorala and Piercey-Normore, 2015). Urease is produced by several lichens (presumably by the photobiont) and is secreted into the culture medium under laboratory conditions (Perezurria et al., 1989, 1993). The secretion of urease into the medium is hypothesised to be the consequence of its transfer from the photobiont to the mycobiont, depending on the nitrogen content of the mycobiont as well as the water content of the lichen thallus (Perezurria et al., 1989). However, it is not clear, whether the secreted ureases play a role similar to that of membrane-bound urease.

The lectin recognition and signalling mechanism summarised for chlorolichens in Figure 2 can be true of all or some cyanolichens (Sacristán et al., 2007; Vivas et al., 2010; Díaz et al., 2016a). Diaz et al. (2015), Diaz et al. (2016b) showed that actin- and myosin-like proteins produced by the cyanobacterial photobiont *Nostoc* of the lichen *Peltigera canina* is involved in the chemotactic movement of photobiont cells towards the lectin of the mycobiont. The process also involves a contractile protein and ATPase of photobiont, which creates a series of contraction-relaxation steps that result in photobiont movement towards mycobiont lectin (Diaz et al., 2011). Upon cell contact of photobiont and mycobiont, desensitisation occurs and photobiont contractile motility stops. It is yet unknown if a similar type of chemotaxis applies to chlorolichens.

It is speculated that mycobionts of some lichens not only rely on lectin-ligand recognition mechanisms for establishing the initial photobiont contact, but that these mechanisms might also be involved in further replication and growth of young photobiont cells within the lichen thallus (Díaz et al., 2016a). The factors triggering the initiation of symbiont recognition through lectin-ligand binding and the ways in which symbionts increase the probability of association have been poorly understood, although several hypotheses have been proposed (Díaz et al., 2016a). For example, the photobiont could secrete a yet unknown diffusible compound that is sensed by a compatible mycobiont to trigger mycobiont lectin biosynthesis. Mycobionts may also produce multiple lectins with competing specificities for different photobionts, which may also be a strategy for rejecting incompatible photobionts. We could test some of these hypotheses using a systems biology approach, for example, through time-course analysis of coupled gene expression and metabolome profiles of lichen co- and mono-cultures to identify candidate genes and molecules with potential signalling roles. Armaleo et al. (2019) recently pursued a transcriptome study exploring the differential expression of genes involved in symbiosis and signalling between *Cladonia grayi* and its algal partner *Asterochloris glomerata.* While only a snapshot in time, this work provided unprecedented insights into the complexity of responses underlying lichen symbioses.

### Exudates Signalling in Lichens

**FIGURE 3:**
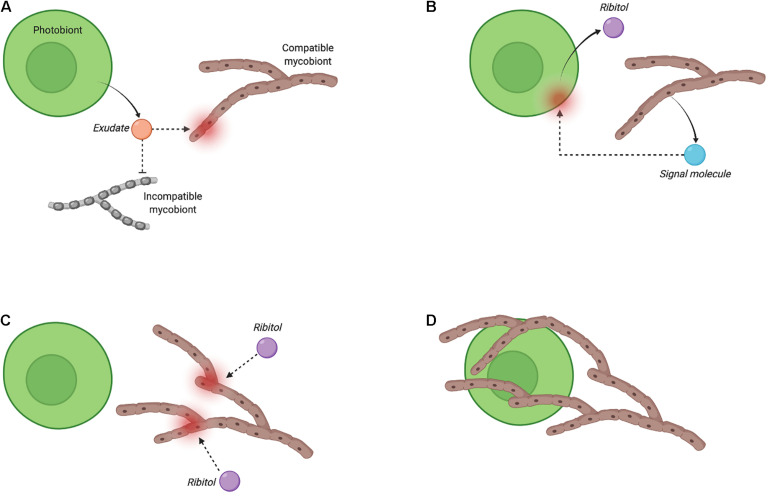
Proposed exudate-based recognition and signalling for *Fulgensia bracteata* (adapted from Meessen et al., 2013). The bi-directional and multi-step recognition process (i.e., pre-contact stage) is initiated by a compatible photobiont through secretion of an exudate, which is speculated to be species-dependent but has yet to be identified for most lichen species **(A)**. The exudate induces the production and secretion by the mycobiont of a yet unknown signalling molecule that stimulates ribitol release by the photobiont **(B)**. Ribitol then triggers fungal growth by relieving hyphal growth arrest **(C)**, and allowing hyphal branching, and the engulfment of the photobiont cell by the mycobiont **(D)**. The two last steps may be accompanied by the secretion of a mucilage in some lichens (Meessen et al., 2013; Meessen and Ott, 2013). (Image created with BioRender.com).

Carbohydrate release and translocation from photobiont to mycobiont of a lichen was first proposed in the mid-1960s by Drew and Smith, who used radioactive isotope tracing to estimate the proportion of labelled carbon in sodium [^14^C]-bicarbonate fixed to [^14^C]-glucose by the cyanobacterial symbiont (*Nostoc*) of *Peltigera polydactyla* compared with its free-living and cultured forms (Drew and Smith, 1967a, b). Carbohydrate movement from photobiont to mycobiont has been investigated for more than 30 additional lichens and is reviewed elsewhere (Smith et al., 1969). The results of these studies showed that glucose and sugar alcohols are the main forms of carbohydrates released by cyanobacterial and microalgal photobionts, respectively, and that they are translocated to the respective mycobionts. In the absence of a symbiotic relationship, the levels of carbohydrate released by the photobionts decline significantly or drop to zero. Following these initial studies, the importance of carbohydrate release by lichen photobionts gained a renewed interest in efforts to uncover the molecular mechanisms behind the early stage of lichenisation (Joneson et al., 2011; Meessen et al., 2013; Meessen and Ott, 2013; Athukorala et al., 2014; Athukorala and Piercey-Normore, 2014; Armaleo et al., 2019). A possible exudate signalling model based on the release and movement of ribitol is shown in Figure 3, and is largely based on independent studies observing ribitol release in the cultures of *Gyalolechia bracteata* (Meessen et al., 2013; Meessen and Ott, 2013) and *Cladonia grayi* (Joneson et al., 2011). Although the exact nature of the secreted molecules in this exudate-signalling model has not been fully elucidated, it is speculated that an exchange of carbon and nitrogen could be the driver for uniting symbionts in the first place. Hom and Murray (Hom and Murray, 2014) showed that co-culturing of model fungi *Saccharomyces cerevisiae, Aspergillus nidulans, or Neurospora crassa* with the alga *Chlamydomonas reinhardtii* could facilitate mutualistic interactions through exchanging carbon and nitrogen under specific growth conditions; their results also suggest that carbon released by mycobiont respiration (as CO_2_) could be recaptured by the photobiont for efficient carbon recycling within the lichen symbiosis (Schwartzman, 2010). Thus, the need for nutrient exchange between species could trigger the initiation of symbiotic interaction in lichens. Signalling network modelling, discussed in the following section, is one approach to generate insights on how specific exudate compounds could play a role in the overall flow of signals through the proposed ‘exudates signalling’ mechanism.

### Signalling Network Modelling: Challenges and Opportunities for the Lichen Symbiosis

A signalling network consists of a series of ‘signals’ and ‘receptors’ whose relationships are determined by the signal transduction mechanisms governing the network. These signals and receptors could be any or combination of enzymes (e.g., kinases), organic substances (e.g., ATP), inorganic molecules (e.g., phosphates), or other proteins or biomolecules. Reactions connecting these molecules frame the underlying signalling mechanisms and the goal of signalling network modelling would be to predict such interactions and the emergent cascade of signalling events that can explain or predict the behaviour of the signalling network. Signalling network models are often divided into descriptive and predictive subtypes. Descriptive models are usually simpler and provide a qualitative overview of the signalling pathway structure (i.e., topology of signal molecules and reactions), whereas predictive models may capture kinetics of the signalling pathway (i.e., reaction rates) and be capable of estimating system behaviours under new perturbations. The application of diverse descriptive and predictive modelling to signalling networks has been reviewed elsewhere (Hyduke and Palsson, 2010; Morris et al., 2010; Terfve and Saez-Rodriguez, 2012; Rother et al., 2013; Lavrik and Samsonova, 2016; Antebi et al., 2017). The scope and choice of signalling network modelling approach vary with the complexity of the network being explored. For example, some of the most detailed and comprehensive predictive signalling models have been developed for complex but known signalling networks of human B-cells (Papin and Palsson, 2004), prostate cancer cells (Dasika et al., 2006; Vardi et al., 2012), and Toll-like receptors (TLRs) functioning in immune system (Li et al., 2009).

In symbiotic systems, signalling pathways have been a topic of particular focus for legumes-rhizobia and plants-root fungi (mycorrhiza) symbioses (Bonfante and Genre, 2010; Bonfante and Requena, 2011; Oldroyd, 2013; Venkateshwaran et al., 2013; Mohanta and Bae, 2015; Martin et al., 2017; Poole et al., 2018; Clear and Hom, 2019). However, modelling the signalling networks in these systems has not received much attention, perhaps due largely to the knowledge gap in certain key signalling steps. For example, in the common symbiotic signalling “SYM” pathway, which shares similar signalling steps between arbuscular mycorrhizal and rhizobial symbioses, it remains unclear how symbiosis receptor kinases (SYMRK) transmit signals to downstream cation channelling proteins (i.e., CASTOR/POLLUX) located in the nucleus (Huisman and Geurts, 2020). Moreover, the precise mechanisms for how plants discriminate between arbuscular mycorrhiza and rhizobia symbionts are still unknown, although signalling pathways functioning in parallel to the SYM seem likely to be involved. Modelling signalling networks could represent a complementary approach to fill such gaps by simulating system behaviours with proposed/candidate mechanisms implemented by which symbionts transduce signals and communicate.

Faced with the paucity of detailed mechanistic knowledge on signalling networks in lichens (despite several potential signal molecules identified; see Table 1), the modelling of signalling networks in lichens suffers from similar challenges as those of other symbiotic systems and no models have yet been reported. Nevertheless, given the recent availability of ‘omics’ data for a variety of lichens (Mittermeier et al., 2015; Wang et al., 2015; Armaleo et al., 2019), there are now new opportunities to develop signalling models of lichens. For instance, a proteomics approach could enable measuring lectin and urease levels of lichen cultures at pre- and post-contact stages informing the relative abundances of these proteins. The proteome profile of such cultures could also indicate the presence/absence of other specific proteins at the corresponding stages of lichenisation that may correlate with lectin/urease activity levels and provide deeper insights into how the recognition process initiates. A signalling pathway model could be developed to explore the link between putrescine biosynthesis and lectin production in repression of cell wall disruption of compatible photobiont as described in Figure 3.

## Metabolic Interplay in the Lichen Symbiosis

**FIGURE 4:**
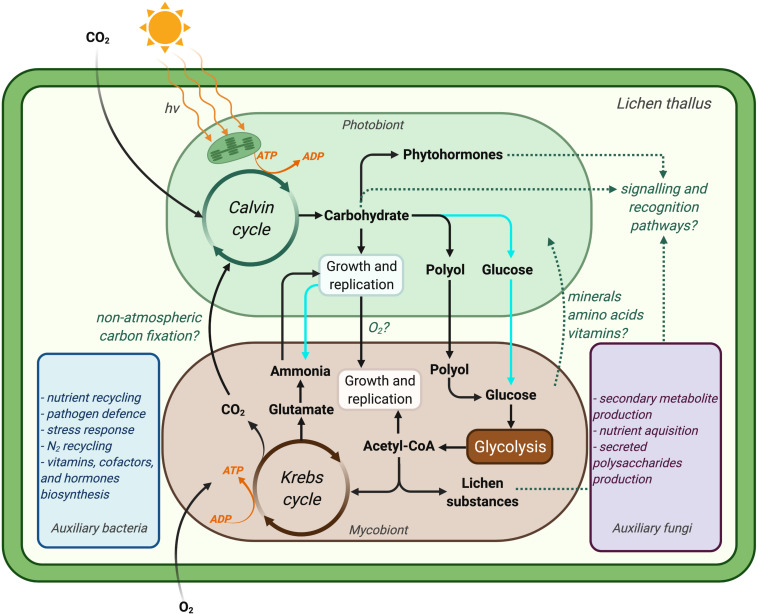
Simplified illustration of a lichen symbiosis showing our current understanding of the metabolic interactions between lichen symbionts. Dotted line indicates knowledge gaps that may not be generalisable to all lichens. Atmospheric carbon is fixed to carbohydrates by the photobiont at the expense of ATP, which is generated by photosynthesis. Chlorolichens convert carbohydrate to polyols and transport it to their respective mycobionts, whereas cyanolichens (cyan pathways) translocate carbohydrate in the form of glucose to their mycobionts. Mycobiont metabolism is fuelled by such carbohydrate translocation, which results in the provisioning of nitrogen for the photobiont (in case for chlorolichens). Recent studies also suggest coupled metabolisms for lichen symbionts with respect to the supply of oxygen (in addition to carbohydrates) by photobionts in return for carbon dioxide from their mycobionts (Schwartzman, 2010; Hom and Murray, 2014; ten Veldhuis et al., 2020). The production of lichen substances (mainly by mycobionts) and the production of phytohormones and/or carbohydrates by photobionts are believed to play key roles in the recognition of symbionts. The proposed functions of auxiliary lichen bacteria and fungi during the symbiosis are also indicated based on comprehensive multi-omics studies (blue and purple rectangles). It is speculated that the lichen microbiome forms a biofilm on the surface of the mycobiont, through which they communicate (Image created with BioRender.com).

The literature on lichen metabolism has been largely focused on understanding the exchange of key nutrients between symbionts (Lines et al., 1989; Kono et al., 2020; ten Veldhuis et al., 2020) and identifying lichen secondary metabolites and their biosynthetic pathways (i.e., metabolite profiling) (Fahselt, 1994; Aubert et al., 2007; Elix and Stocker-Worgotter, 2008; Mittermeier et al., 2015; Bertrand et al., 2018b; Brakni et al., 2018; Calcott et al., 2018; Kuhn et al., 2019; Goga et al., 2020; Figure 4). In the 1960s, observations of carbohydrate storage and translocation between the symbionts of *Peltigera polydactyla* (Smith and Drew, 1965; Drew and Smith, 1967a, b) together with a series of similar studies on other lichens (Smith et al., 1969) established the foundations for studying the metabolic interplay in lichens. The primary aim of those studies was to identify the form of carbon translocated between lichen symbionts, as explained in the previous sections. Next to nothing is known about the metabolic program and gene expression in lichen symbionts following carbohydrate exchange and assimilation. Most metabolic studies in lichens have concentrated on understanding the overall carbon and nitrogen economy in lichens, mainly with respect to overall carbon fixation, carbon sinks, lichen growth, and nitrogen fixation by cyanolichens (Honegger et al., 1993; Dahlman et al., 2004; Nash, 2008b; Palmqvist et al., 2008). Eisenreich and colleagues (Eisenreich et al., 2011) suggested that using ‘omics’ methods together with isotope labelling experiments (increasingly referred to as ‘fluxomics’) could enhance our understanding of lichen metabolic pathways, although this has yet to be fully realised to study lichen metabolism at a systems-level.

Thus, despite of being broadly recognised that carbohydrates and inorganic molecules are exchanged between lichen symbionts, a systems-level molecular understanding of metabolism is still lacking for lichens, including their primary symbionts and auxiliary partners. This lack has left key features of metabolism unexplored, including, for example, central aspects of carbon metabolism with respect to lichen compartmentalisation or the role of cell wall components and biosynthesis on the growth and metabolite exchange between symbionts. A systems-level understanding of lichen metabolism will become more likely in near future in light of the recent insights on lichen microbiota composition and functions within the lichen symbiosis (Spribille et al., 2016; Cernava et al., 2017; Smith et al., 2020).

Rhizobiales have been found to be a dominant bacterial order in the microbiome of various terrestrial or marine lichens examined to date (Grube et al., 2009; Hodkinson and Lutzoni, 2009; Hodkinson et al., 2012; Erlacher et al., 2015). Specifically, Rhodospirillales were found to be common in chlorolichens, and Sphingomonadales and Bacteroidetes in cyanolichens (Hodkinson et al., 2012; Graham et al., 2018; West et al., 2018). Several factors are believed to influence lichen-associated bacterial community composition and diversity. These include the nature of lichen secondary metabolites (driven mainly by the type of primary mycobiont), large-scale geography, growth type, and the type of primary lichen photobiont (Grube et al., 2009; Hodkinson et al., 2012; Aschenbrenner et al., 2016). Some of these auxiliary bacteria were thought to be able to fix atmospheric nitrogen and, as cyanobacterial photobionts, might play an important role as a nitrogen source for the lichen symbiosis (Hodkinson and Lutzoni, 2009). Additionally, cyanobacterial lichens, which often grow in nitrogen-limited environments, were shown to harbour a diversity of bacteria that would otherwise not grow in such nitrogen-limited environments (Hodkinson et al., 2012). Apart from nitrogen fixation, meta-omics (e.g., meta-genomics, meta-transcriptomics, and meta-proteomics) studies have revealed functional roles for the microbiome of the lichen *Lobaria pulmonaria*, including: nutrient recycling in the decaying parts of the lichen thallus, pathogen defence, detoxification processes, protection against oxidative stress, biosynthesis of vitamins, cofactors, and hormones, activation of ketone metabolism during dehydration, and upregulated transcription of transport systems, tRNA modification and various porins during hydration (Cernava et al., 2015; Grube et al., 2015; Aschenbrenner et al., 2016; Sigurbjornsdottir et al., 2016; Cernava et al., 2017; Cernava et al., 2019). The role of these auxiliary bacteria is thus critical to the maintenance and functioning of the lichen symbiosis.

The large diversity of lichen-associated fungi has been revealed through culture-dependent methods first (Petrini et al., 1990; Arnold et al., 2009), then meta-omics data analyses (Spribille et al., 2016; Smith et al., 2020). The low biomass of these auxiliary fungi relative to the primary mycobiont and the inability to culture them have prevented a detailed analysis of their functional roles in the lichen symbiosis. However, based on the analysis of meta-genome of the lichen *Alectoria sarmentosa*, a recent study showed that auxiliary fungi (two basidiomycete yeasts) may play roles in producing secreted extracellular polysaccharides, lichen nutrient acquisition, and secondary metabolite production (Tagirdzhanova et al., 2021). They are therefore also likely to play an important role in the maintenance and functioning of the lichen symbiosis.

Although meta-omics analyses of lichen microbiomes have provided invaluable insights on the diversity and function of multi-species lichen symbioses, constraint-based metabolic modelling could potentially enable a deeper understanding of the multi-species metabolic interplay. For example, by applying a systems biology approach using genome-scale metabolic reconstructions for 773 human gut bacteria (AGORA), a more sophisticated understanding of the interactions between the host and gut microbiome was achieved, revealing how system responses depended upon the metabolic potential of each component species and the nutrients available (Magnusdottir et al., 2017). The AGORA framework confirmed that a high fibre diet (usually linked to a healthy microbiome) would result in higher proportion of commensal and mutualistic pair-wise interactions between gut microbes. This framework was able to show how the host-microbiome operates mechanistically and indicate how many positive interactions are sufficient to maintain a healthy gut community. A similar systems-level understanding of lichens could help in understanding the metabolic interdependency for symbiotic establishment and maintenance, and in predicting the role of associated lichen microbes and lichen responses to environmental changes or likely environmental niches. This would also aid in re-creating/re-synthesizing lichens *in vitro* and using them for biotechnological applications.

### Genome-Scale Metabolic Flux Modelling: Challenges and Opportunities for the Lichen Symbiosis

Genome-scale metabolic network models simulate the metabolism of a living cell as a collection of hundreds to thousands of biochemical reactions (forming metabolic pathways of an organism) and enable quantitative and gene-grounded predictions of phenotypes under different growth conditions (Varma and Palsson, 1994; Covert et al., 2001). This set of reactions is framed as a set of ordinary differential equations, in which the number of variables and equations are defined by the number of metabolites and reactions, respectively. Solving this system of equations under a given set of assumptions (e.g., net zero system flux or “flux balance”) allows for determining optimal fluxes for each reaction in the metabolic network. Specific constraints describing the physico-chemical, environmental, regulatory, and/or topological conditions of the metabolic network can be imposed to identify optimal flux distributions consistent with these assumptions (Price et al., 2004). Such constraint-based metabolic modelling enables a wide range of applications including, but not limited to, predicting cellular functions (e.g., energy production) (Edwards et al., 2001; Orth and Palsson, 2012; Bordbar et al., 2014), identifying optimal strains and culture media conditions for specific applications (Pharkya et al., 2004; Nazem-Bokaee and Senger, 2015), formulating metabolic/strain engineering strategies (Burgard et al., 2003; Chung et al., 2010; Kim and Reed, 2010; Ranganathan et al., 2010; Rocha et al., 2010; McAnulty et al., 2012; Yen et al., 2013; Kim et al., 2019), identifying drug targets (Kim et al., 2011, 2012; Angione, 2019; Gu et al., 2019), producing natural/non-natural chemicals and precursors (Yim et al., 2011; Ye et al., 2014; Nazem-Bokaee et al., 2016; Wei et al., 2017; Nazem-Bokaee and Maranas, 2018; Biz et al., 2019; Gu et al., 2019), creating knowledgebases of metabolic, genomic, and biodiversity information (Kumar et al., 2012; Pabinger et al., 2014; King et al., 2016; Nazem-Bokaee et al., 2017; Norsigian et al., 2020), and studying syntrophic/symbiotic communities (see below). Table 2 lists select examples of two-species metabolic models that have been studied.

**TABLE 2:** Select two-species metabolic network models that have been constructed and analysed^1^.

Partners/symbionts^2^	Community modelling approach^3^	Key outcomes of the study	References
*Desulfovibrio vulgaris* (r: 89) *Methanococcus maripaludi* (r: 82)	Compartmentalised; steady-state	This is the first study on modelling mutualistic interactions between a sulphate-reducing bacterium and a methanogen using a compartmentalised approach. Using relatively small metabolic networks of the two microbes, a syntrophic methanogenesis was simulated when *D. vulgaris* produced hydrogen, carbon dioxide, and acetate, which were utilised by the methanogen.	Stolyar et al., 2007
*Geobacter sulfurreducens* (c: 2, g: 588, r: 727) *Rhodoferax ferrireducens* (c: 2, g: 744, r: 762)	Compartmentalised; dynamic	This work analysed the dynamics of growth between two bacteria competing for uranium bioremediation.	Zhuang et al., 2011
*Scheffersomyces stipites* (c: 3, g: 814, r: 1371) *Saccharomyces cerevisiae* (c: 8, g: 904, r: 1412)	Lumped; dynamic (s: 3588)	In this study a co-culture simulating lignocellulosic feed breakdown for biofuel production was analysed using metabolic models of *S. cerevisiae* converting hexose and *S. stipites* converting pentose part of the synthetic feed into ethanol.	Hanly and Henson, 2013
*Geobacter metallireducens* (c: 2, g: 987, r: 1284) *Geobacter sulfurreducens* (c: 2, g: 837, r: 1085)	Compartmentalised; steady-state (t: 36)	A multi-omics approach was used in this study to understand electron flow mechanisms between the two bacteria. Results suggested that while *G. metallireducens* could respond only to syntrophic changes at transcriptomic level, *G. sulfurreducens* responded at both transcriptomic and genomic levels.	Nagarajan et al., 2013
*Bifidobacterium adolescentis* (g: 452, r: 699) *Faecalibacterium prausnitzii* (g: 484, r: 713)	Compartmentalised, steady-state	This study demonstrated that through modelling only two representatives of human gut microbiome, *B. adolescentis* and *F. prausnitzii*, the growth of the latter is severely affected when acetate production by the first microbe became limited.	El-Semman et al., 2014
*Salmonella enterica Escherichia coli K12 strain*	Compartmentalised; dynamic	Community modelling confirmed growth of *E. coli* on lactose minimal media was feasible only in co-culture with *S. enterica*, which received acetate and produced methionine in return.	Harcombe et al., 2014
*Escherichia coli K strain* (c: 3, g: 1260, r: 2073) *Escherichia coli L strain* (c: 3, g: 1260, r: 2073)	Compartmentalised; dynamic (t: 2)	Auxotrophy was studied using two mutants of *E. coli*, in which one grew with leucine and produced lysine that was assimilated by the other strain.	Zhang and Reed, 2014
*Ketogulonicigenium vulgare* (c: 3, g: 663, r: 2073) *Bacillus megaterium* (c: 3, g: 1055, r: 2073)	Compartmentalised; steady-state (t: 453)	In this study an artificial consortium was constructed to analyse the production of vitamin C and other metabolites (e.g., 2-keto-l-gulonic acid) during two-step fermentation process	Ye et al., 2014
*Leptospirillum ferriphilum* (r: 87) *Ferroplasma acidiphilum* (r: 71)	Compartmentalised; steady-state	In this work, a bacteria-archaea mixed culture was modelled to study bioleaching (oxidizing iron)	Merino et al., 2015
*Chlamydomonas reinhardtii* (c: 10, g: 1080, r: 2191) *Saccharomyces cerevisiae* (c: 8, g: 750, r: 1266)	Compartmentalised; dynamic (t: 2)	The goal of this study was to feed process models with metabolic models of algal-fungal co-culture for optimizing biodiesel production. The alga produced oxygen for the yeast and in return received carbon dioxide secreted by the yeast. This study is an example of creating artificial symbiosis through exchange of key metabolites between an alga and a fungus, which could lead to higher biodiesel production compared with single cultures of the alga.	Gomez et al., 2016
*Thermosynechococcus elongatus BP-1* (g: 583, r: 917) *Meiothermus ruber strain A* (g: 729, r: 1163)	Lumped and compartmentalised; steady-state (s: 1707)	The lumped model showed highest overall consistency between predicted fluxes and measured gene expression data. However, this approach provided no information on the potential interactions between the two members of consortia. The gap-filled compartmentalised model provided the best performance among all models with respect to predicting key metabolites interacting between the two bacteria.	Henry et al., 2016
*Medicago truncatula* (c: 8, g: 3403, r: 2909) *Sinorhizobium meliloti*	Compartmentalised; steady-state (t: 20)	The community model predicted the preferred uptake of ammonia over nitrate when both present in excess. At dark and when ammonia is limiting, the model predictions were in favour of nitrate uptake. The symbiotic model predicted amino acid cycling which is shown to be essential for nitrogen fixation for some rhizobial strains.	Pfau et al., 2018
*Nitrosomonas europaea* (g: 578) *Nitrobacter winogradskyi* (g: 579)	Compartmentalised; dynamic (t: 25)	Aerobic co-culture of two model nitrifying bacteria was used to study the dynamics of nitrification in agricultural settings	Mellbye et al., 2018
*Phaeodactylum tricornutum* (c: 6, g: 1027, r: 4456) *Pseudoalteromonas haloplanktis* (c: 2, g: 721, r: 1322)	Lumped; dynamic (s: 3588)	This work demonstrates the advantages of using metabolic models to simulate a diatom-bacteria co-culture to study the effect of changes in growth parameters on the co-culture to represent ocean food ecosystem. Using a linear community-level biomass objective function, a multi-compartment model was built, and then, converted into a dynamic, constraint-based, model of co-culture. Simulating this synthetic ecosystem revealed that the growth of the diatom was negatively affected by the growth of the bacterium due to the shortage of phosphate and sulphate.	Fondi and Di Patti, 2019

*^1^ Community metabolic models developed to study interactions among more than two organisms in any microbiota was excluded in this table for simplicity. For further information on larger communities of microbes the reader is referred to the text and these reviews (Zomorrodi and Segre, 2016; Ang et al., 2018; Chan et al., 2017a; Gu et al., 2019). ^2^ Numbers in parenthesis indicate the number of compartments (c), genes (g), and reactions (r), if available, captured in the respective metabolic model of the symbiont. ^3^ Numbers in parenthesis indicate the number of inter-species transporters (t) or shared reactions (s), when available, captured in the respective community metabolic model.*

Techniques developed for the characterisation of metabolic interactions among members of microbial communities based on genome-scale metabolic modelling can be classified into two main groups: lumped (also called enzyme soup, mixed bag, or metagenome-scale modelling (Chan et al., 2017a)) and compartmentalised (Biggs et al., 2015; Henry et al., 2016; Zomorrodi and Segre, 2016). The analysis of interactions in a microbial community can be performed under steady-state or dynamic conditions. While an extensive description of these techniques and their implementation can be found elsewhere (Biggs et al., 2015; Zomorrodi and Segre, 2016; Chan et al., 2017a; Ang et al., 2018; Garcia-Jimenez et al., 2021) and is beyond the scope of this review, it is worth broadly covering the general aim of each technique. The lumped modelling approach seeks to find optimal conditions that benefits the whole community (e.g., mutualistic symbiosis) by neglecting boundaries between members of the community (Taffs et al., 2009; Henry et al., 2016). The compartmentalised modelling approach, on the other hand, retains boundaries between members while also allowing individual members to share a compartment and transfer metabolites. For example, the compartmentalised modelling approach enables considering a member-level objective towards achieving a community-level objective by imposing a constant growth rate across all members for a community to ensure co-existence and stability (Chan et al., 2017b). Although computationally more expensive, the compartmentalised modelling approach also allows for the study of different types of species-species interactions (e.g., parasitism) (Zomorrodi and Maranas, 2012). A dynamic modelling approach enables predictions of changes in metabolites and biomass over time within the community and relies on kinetic data of uptake reactions. The dynamic approach has been extended to enable spatial analysis of communities, as in the COMETS (Computation Of Microbial Ecosystems in Time and Space) framework, which coupled metabolic with diffusion modelling and was applied to understand metabolite exchange within a three-member microbial community (Harcombe et al., 2014).

To our knowledge, no genome-scale metabolic network model has yet been constructed for any lichen association or its symbionts. With the first genomes of mycobionts (Park et al., 2013a, b, 2014a, b; Armstrong et al., 2018; Bertrand et al., 2018a; Wang et al., 2018) and photobionts (Armaleo et al., 2019) of several lichens assembled and more foreseen to come, it is a timely opportunity to understand the lichen symbiosis through the lens of genome-scale metabolic models. Since little is known about the metabolic response of lichens to different environmental conditions (e.g., light intensity, water content, nutrient availability, etc.), developing a metabolic network model could shed invaluable insights on symbiosis at the molecular level. Furthermore, the available computational tools for modelling community interactions could allow for predicting the role of a specific symbiont on the performance of a lichen under a known environmental perturbation (e.g., nutrient limitation). A lichen metabolic model could be used as the framework for the integration of ‘omics’ data obtained for lichens to test multiple hypotheses including, for example, the regulatory effect of different carbohydrates on the growth and exchange of metabolites between lichen symbiont. Since *in vitro* lichen re-synthesis is still hampered by the complexity of the lichenisation process, metabolic modelling could highlight potential metabolites that may need to be exchanged between symbionts as well as the metabolic pathways that may lead to successful differentiation and growth. Moreover, metabolic modelling could be used to examine the potential for symbiosis between various combinations of mycobionts and photobionts, and provide insights into the evolution of the lichen symbiosis. Validating predictions of flux distribution by community metabolic models could be a challenge, due to multi-compartmental nature of lichen symbiosis and difficulties in measuring fluxes through each compartment *in vivo*. However, recent advances in the field of metabolic flux analysis now make it possible to resolve fluxes by carefully designing the isotope labels and tracing them across different compartments (Schwechheimer et al., 2018). Another practical challenge for the development of lichen metabolic models may pertain to the characterisation of the cellular composition of individual lichen symbionts. For example, many lichen mycobionts grow slowly, making it experimentally difficult to obtain sufficient cell mass needed to formulate a ‘biomass’ reaction in a metabolic model representing cellular growth. Moreover, due to the lack of data specific to the metabolic pathways of lichens, the model curation process may be patchy, with irreconcilable gaps and network disconnects. However, metabolic models for lichens could be reconstructed by leveraging the ever-increasing number of high-quality metabolic models becoming available for not-too-distantly related filamentous fungi, microalgae, or cyanobacteria (Brandl and Andersen, 2015; Gomez et al., 2016; Santos-Merino et al., 2019).

## Conclusion and Future Perspectives

Lichens, although historically well-known and iconic symbioses, still bear a sense of mystery as our understanding of the signalling networks and pathways responsible for their symbiotic establishment and maintenance is still in its infancy. Two signalling mechanisms were reviewed in this article but many more could be explored with the aid of techniques such as untargeted metabolomics. Signalling/metabolic network modelling approaches could support the field of experimental lichenology by providing insights into: (1) the signalling molecules and the roles they play at different stages of lichenisation, (2) how lichen symbionts benefit from the symbiosis with regards to carbon, nitrogen, and other limiting nutrients or environmental conditions, (3) which conditions allow lichens to produce secondary metabolites and the genes that are involved, and (4) how lichens manage to accumulate and tolerate high levels of toxic metals. Advances in DNA sequencing technologies in recent years have significantly reduced the cost of generating genome sequences. At the same time, improvements in high performance computing and development of more biologist-friendly tools for modelling and analysing ‘genome-scale’ metabolic networks have enabled the exploration of metabolically-coupled microbial communities. Combining these genome resources and systems biology tools could open up a whole new era for the study of the lichen symbiosis.

## Author Contributions

HN-B and CG conceptualised and wrote the manuscript. HN-B and CG designed and created figures. EFYH, ACW, and SM revised the manuscript. All authors read and approved the final manuscript.

## Conflict of Interest

The authors declare that the research was conducted in the absence of any commercial or financial relationships that could be construed as a potential conflict of interest.
